# Expectation-[in]congruence differentially impacts recall and recognition of object features

**DOI:** 10.3758/s13421-025-01740-x

**Published:** 2025-06-10

**Authors:** Kimele Persaud, Carla Macias, Elizabeth Bonawitz

**Affiliations:** 1 Department of Psychology, Rutgers University–Newark, Newark, NJ USA; 2 Graduate School of Education, Harvard University, Cambridge, MA USA

**Keywords:** Memory, Object-feature, Prior expectations, Color, Congruency effects, Recall, Recognition, Cognition

## Abstract

Study events that are congruent with our prior expectations are better remembered than expectation-unrelated events. Paradoxically, events that are highly incongruent with expectations are also better remembered. In this study, we explore whether this paradoxical finding persists in object featural memory. Specifically, we examine whether memory for expectation-congruent and incongruent features of objects is differentially impacted by the processes that underlie recall and recognition and the types of information being probed. In three experiments, we manipulated the degree to which object features adhered to people’s prior expectations (i.e., colors of objects) and then assessed memory (recall and recognition) for expectation-relevant features (i.e., object–color) and expectation-irrelevant features (i.e., object-shape). While both expectation-congruent and incongruent features were equally well recognized, only expectation-congruent features were better recalled compared to expectation-unrelated features. Furthermore, only strong expectation-congruence created a memory advantage for expectation-irrelevant object features. These findings suggest that in object featural memory, expectation-congruence and incongruence are qualitatively dissociable in their impact on recognition and recall processes. The findings from this work have important implications for cognitive and neuroscientific theories of how prior expectations shape the representation of objects and their constituent features in episodic memory.

**Supplementary Information:**

The online version contains supplementary material available at 10.3758/s13421-025-01740-x.

## Introduction

Our expectations about the world and our episodic memory for past experiences are intricately linked, but this relationship is highly complex. The presence and strength of our expectations differently dictate the kinds of information that get prioritized in memory. For example, past episodes that are congruent with our prior expectations are often better remembered than incongruent or unrelated episodes (e.g., Hemmer & Persaud, 2014; Hemmer & Steyvers, 2009; Persaud & Hemmer, 2014). For instance, we might have good memory for what we ate for breakfast last Monday or what we wore to the last football game if these events match what we usually eat or what we usually wear at sporting events. At the same time, past episodes that are expectation-incongruent are also better remembered (e.g., Greve et al., 2017; Sakamoto & Love, 2004; Stahl & Feigenson, 2017). For instance, we would also remember if last Monday we felt adventurous and ate a burger for breakfast as opposed to the usual bowl of cereal. As such, a long-standing question is what mechanisms underlie these potentially conflicting findings. That is, what are the context dependencies that govern when one type of expectation-related information is better remembered than the other? The aim of the present study was to bring together these lines of research to investigate the impact of expectation-congruence on episodic memory for the intrinsic properties (e.g., color, shape) of visual objects and identify potential factors that might dissociate expectation-congruent versus expectation-incongruent object feature memories. Investigating the influence of expectation-congruence on object feature memory is crucial for understanding how objects, critical building blocks of past episodes, are represented and recalled from visual memory, and for disentangling the factors that influence the relationship between prior expectations and episodic memory, more broadly.

### Expectation-congruence and memory

A prevailing finding in the literature is enhanced memory for information that is congruent with prior knowledge and expectations about the world compared to expectation-incongruent or expectation-unrelated information (e.g., Hemmer & Persaud, 2014; Hemmer & Steyvers, 2009; Persaud & Hemmer, 2024). This congruency effect has been demonstrated across a broad range of stimuli domains, including memory for object–location pairs (Quent et al., 2022; van Kesteren et al., 2014), object–scene pairs (Brod & Shing, 2019; van Kesteren et al., 2013), word pairs (Alejandro et al., 2021), and word–phrase pairs (Bein et al., 2015; Höltje et al., 2019).

In these contexts, prior knowledge and expectations, in the form of schemas, are thought to influence the retrieval of information from memory (Bartlett, 1932; Brewer & Treyens, 1981; Persaud & Hemmer, 2024). For example, in a classic study, Brewer and Treyens (1981) investigated the role of room schemas on recall and recognition of objects in a real-world room (e.g., graduate student office). They found that schema knowledge of scenes enhanced recall and recognition of objects congruent with the schema relative to objects incongruent with the schema. It also produced intrusion errors whereby participants falsely reported objects that were congruent with the schema, but not actually present in the room (i.e., books). A similar intrusion error was found in studies using word lists whereby participants falsely recalled semantic associates to words on a studied list that were not actually present on the list (Deese, 1959; Roediger & McDermott, 1995). Taken together, these studies suggest that expectation-congruent information benefits from enhanced memory over incongruent information due to integration with pre-existing knowledge in the form of schemas.

One of the earliest theories to explain this congruent advantage was Craik and Tulving’s (1975) *integration-elaboration* theory. According to this theory, when a target item is presented in an expectation-congruent context, it is more easily integrated, leading to a more elaborate memory trace that is less effortful to retrieve later on. This work was recently extended to show that the memory advantage for the congruent condition applies not only to the congruent target item but also to the congruent context (Bein et al., 2015). For example, in an incidental learning task, Bein and colleagues (2015) showed participants target words that were presented with either congruent or incongruent context word pairs and found enhanced memory not only for target words presented in congruent pairs but also better retrieval of the context words associated with the recollected target words compared with the incongruent condition. This suggests that participants were able to integrate the target and context word into an elaborate memory trace that subsequently promoted better retrieval of both words.

A similar account of why expectation-congruent information is better remembered, specifically during recall tasks, is that prior expectations can serve as a retrieval cue for searching memory. That is, using prior expectations as cues to search memory is more likely to tag and retrieve congruent compared with incongruent information, therefore resulting in better recall of expectation-congruent information (Sherman & Frost, 2000). Prior knowledge also helps to ascribe meaning to study information, further assisting the storage and retrieval of this information in memory (Bransford & Johnson, 1972). Importantly, the enhancement of expectation-congruent items over incongruent or unrelated items in memory relies not only on their relationship to relevant prior knowledge but also on whether the relevant knowledge is activated to help facilitate encoding of congruent items. For instance, Bransford and Johnson (1972) found better memory for the details of vague story prose when participants were informed of the semantic theme of the prose prior to encoding relative to memory for the same prose when no theme was given. This idea of the importance of activating prior knowledge to scaffold memory was further captured by Brod’s (2021) taxonomy of prior knowledge. Therefore, an important contingency for observing better memory for congruent items is whether participants scaffold that knowledge in the context of their prior expectations.

### Expectation-incongruence and memory

An equally prevailing and seemingly conflicting result to the finding that memory is enhanced for information that is congruent with expectations is the finding that memory is also enhanced for information that is highly *incongruent* with expectations relative to no congruence and sometimes expectation-congruent information (e.g., Greve et al., 2017; Sakamoto & Love, 2004; Stahl & Feigenson, 2017). This finding too has been demonstrated across a wide array of stimuli domains, including object–color pairs (Cycowicz et al., 2008; Morita & Kambara, 2022), scene–word pairs (Greve et al., 2017); violations of social stereotypes (Sherman & Frost, 2000), violations of category or schema rules (Greve et al., 2019; Sakamoto & Love, 2004), and violation of object physical properties (Stahl & Feigenson, 2017). For example, when investigating memory for social information, Sherman and Frost (2000) found that participants had better recognition memory for behaviors that violated a stereotype (e.g., a priest who shoved his way to the center seat) versus behaviors that were consistent with a stereotype (e.g., a priest who gave a stranger a quarter).

Verguts and Notebaert (2009) posited the adaptation-by-binding account to explain better memory for expectation-incongruent information. According to this account, conflicting representations, such as expectation-incongruent information, trigger learning mechanisms that selectively guide attention toward violating information. This increased attention leads to enhanced encoding of the target stimuli, which in turn facilitates better memory for the conflicting stimuli at later tests. This same boost in memory is observed when an individual makes an incorrect prediction based on their prior expectation (Brod et al., 2018). That is, when an observer uses their prior expectation to make a prediction, they are surprised when they learn that the correct answer is incongruent with their expectations. This surprise response in turn leads to better memory for the expectation-incongruent information, and this has been argued to be an important factor in learning (e.g., Colantonio et al., 2023, 2024). While there is clear empirical evidence in support of both better memory for congruent and incongruent information, far less work has sought to reconcile these disparate findings and assess the degree to which they impact memory for the intrinsic properties of visual objects.

### Reconciling expectation-congruence, incongruence, and memory

Recent studies have sought to explain these conflicting findings within a single unified framework, with much of this work coming from the domain of cognitive neuroscience. One such framework is the schema-linked interactions between the medial temporal lobe and medial prefrontal cortex (SLIMM model: Greve et al., 2019; van Kesteren et al., 2012). The basis of the SLIMM model is that different brain systems support memory for highly congruent and highly incongruent items. Congruent items are associated with rapid consolidation in memory, and this process involves activity in the medial prefrontal cortex. Whereas items that are highly incongruent with prior expectations can produce a prediction error that triggers activation of structures in the medial temporal lobe (e.g., the hippocampus) and can lead to well encoded memories. Based on the involvement of different brain regions, SLIMM makes several behavioral predictions about memory for expectation-congruent and incongruent information. Here, we bring this model to bear in behavioral predictions of adult memory in a visual feature memory task.

First, SLIMM predicts a nonlinear relationship (e.g., U-shape function) between congruence and memory, whereby items that are highly expectation-congruent *and* incongruent will benefit from enhanced memory relative to items that are less (in)congruent or unassociated with expectations and fall in other locations along a congruence continuum. While a large number of studies compare memory for two congruence conditions, highly congruent and incongruent, far fewer studies have included a third “weak” or less (in)congruent condition to test this U-shaped prediction (e.g., Greve et al., 2017; Lew & Howe, 2017; Quent et al., 2022; van Kesteren et al., 2013). Of the limited studies that have included a weakly (in)congruent condition, the memory results have been mixed. For example, Greve and colleagues (2017), tested memory for highly congruent, less congruent, and highly incongruent items using a schema-based event memory paradigm, and found evidence in support of the U-shaped memory prediction of SLIMM. In contrast, van Kesteren and colleagues (2013) also included a less congruent condition using a scene–word pair memory paradigm and failed to find evidence of the U-shaped memory. Instead, they found better memory for highly congruent items, followed by less congruent items, and finally highly incongruent items. Further, much of this work has examined (in)congruency effects in the context of associative memory. That is, they assess the role of congruence on memory for objects and their associated backgrounds or objects with other objects, and their spatial locations in scenes. As such, it remains unclear how congruence impacts memory for the intrinsic features of objects. In the current study, we include a novel weakly congruent condition where we present objects with less congruent object features to explore the U-shaped function predicted by the SLIMM account. Although the current evidence is limited, we might expect to observe a U-shaped pattern of object-feature memory as a function of congruence.

Second, SLIMM predicts that the enhanced memory for the two extreme conditions of expectation congruence might be influenced by the nature of the retrieval test. That is, while recognition tests might reveal enhanced memory for both highly congruent and incongruent items compared with less or no congruence, recall tests may only reveal better memory for congruent items due to memory search strategies aimed at tagging expectation congruent information (see Sherman & Frost, 2000; Watkins & Gardiner, 1979, for similar arguments). It remains unclear, however, how congruence shapes recall and recognition of object features. Studies that have directly examined how congruence shapes object-feature memory either assess only recognition memory (e.g., object color; Cycowicz et al., 2008), finding better recognition of objects with incongruent feature pairs. Or they assess recall of the object names and not the object feature directly related to congruence (e.g., Morita & Kambara, 2022). As such, it remains unclear whether differences in object feature memory as a function of congruence will be differentially impacted by recall versus recognition processes. It is important to assess both recall and recognition of object features, not only to test predictions of existing accounts but also for a comprehensive understanding of how objects and their constituent features are represented and retrieved from episodic memory. In this investigation, we assess both recall and recognition to capture the influence of expectation-congruence on episodic memory. In line with SLIMM, we predict enhanced memory for highly congruent and incongruent items, relative to less congruent items, with the caveat that memory for incongruent items might be masked in recall, where search processes are more likely to favor the retrieval of congruent information.

Third, the SLIMM account predicts that the enhanced memory for expectation- incongruent items over less or no congruence items will extend to memory for expectation-irrelevant features of that study event due to the involvement of the medial temporal lobe, whereas enhanced memory for expectation-congruent items will not extend to other event features. That is, encountering an expectation-incongruent event is thought to trigger the medial temporal lobe to record a snapshot of the entire event in memory as additional event details might help explain the incongruent aspect of the event and update existing event beliefs. In contrast, congruent events are less likely to trigger the medial temporal lobe as the event details are consistent with existing expectations and therefore can be readily integrated with existing knowledge, in a process managed by the medial prefrontal cortex. To evaluate this prediction, here we test memory for both object features that vary in congruence (i.e., object color) with presented objects as well other intrinsic features that are unrelated to congruence (i.e., object shape).

Despite the existing literature, open questions regarding whether and how expectation-congruence impacts the storage and binding of objects *and* their intrinsic featural properties (e.g., color, shape) in memory persist. As mentioned above, much of the past research examining memory as a function of expectation congruence explores memory for objects and associated elements such as objects paired with backgrounds, objects and spatial locations, word pairs, and words in sentence context. As such the degree to which object-feature congruence shapes memory remains unclear. Furthermore, an open debate in the current literature regards how object representations are stored in memory with some work suggesting that objects and their subsequent features are stored as bound representations (Balaban et al., 2020), while other work suggests that they are stored independently whereby an individual might remember an object but forget its feature (Utochkin & Brady, 2020). It is likely that the stored representation of objects and features (whether bound or unbound) may be impacted by whether or not the feature is expectation-congruent, incongruent, or irrelevant to expectations about the object. Objects are the building blocks of event memory. As such, understanding how objects and their associated features are represented in memory and are influenced by expectation-congruence has important implications for understanding the representations that make up episodic memory.

### Present studies

In three experiments, we sought to evaluate predictions of SLIMM in the domain of object featural memory. We predicted that object featural memory would produce patterns consistent with the predictions of the SLIMM account. Specifically, in Study 1, we first explored whether the U-shaped congruency effect—that is, better memory for highly congruent and highly incongruent items (Greve et al., 2019)—extends to recall for the perceptual features of objects (i.e., color). In Study 2, we explored the impact of congruency on recognition processes and predicted that both highly congruent and highly incongruent items would be better recognized relative to items with less congruent associations. In Study 3, we explored whether the potential boost in memory for expectation-related item features (i.e., object–color) extends to memory for expectation-irrelevant features (object-shape), with the prediction that expectation-irrelevant features would be better remembered when presented with expectation-incongruent objects compared with expectation-congruent and less congruent items.

### Study 1: Cued-recall of object–color pairs

Study 1 examined the effect of object–color congruency on recall processes. Specifically, we manipulated the degree to which the color of a studied object was congruent with people’s expectations (of that object’s color) by introducing a novel “weakly congruent” manipulation and then assessed memory of the object color using a cued-recall task. The object–color pairs varied in two important ways. First, some of the objects had strong a priori expectations for what the color should be (e.g., bananas should be yellow, stop signs should be red), and other objects did not have strong color expectations (e.g., fish, shirt, can be many assorted colors). For objects with strong a priori color expectations, we varied whether the object colors were strongly congruent, weakly congruent, or strongly incongruent with people’s prior expectations. Consistent with the SLIMM account, we predicted better memory for strong congruent and strong incongruent items compared with weak and no congruence items.

### Method

#### Participants

Forty-eight Psychology undergraduate students at Rutgers University–Newark participated for course credit as compensation. All participants provided self-reports of normal color vision. A sensitivity analysis using G*Power software revealed that a within-subjects design with 48 participants across four object–color conditions yields 90% power (alpha = 0.05) to detect effects as small as η_p_^2^ = 0.09 (*f* = 0.32). All participants were run in the laboratory and gave written informed consent prior to the start of the study. At the end of the study, participants were invited to ask the experimenter any questions they had about the study. This study was approved by Rutgers University–Newark Institutional Review Board.

#### Stimuli

The stimuli consisted of line drawings of 27 objects: 10 natural objects (e.g., fruits, vegetables), eight man-made objects (e.g., stop signs, clothes), and nine popular cartoon characters (e.g., SpongeBob, Elmo). Google image search was used to find free-use images. The objects were chosen such that some had strong a priori object–color expectations (e.g., yellow bananas, red Elmo), and other objects did not have strong color expectations (e.g., fish, shirt can be many assorted colors). Second, the expected colors of the objects used spanned seven basic color categories (red, orange, yellow, green, blue, purple, and pink). The objects and their associated colors were learned from two pilot studies with separate groups of participants (*N* = 41 total). In the pilot studies, participants were shown each object one at a time and were asked if they knew the object, and to indicate the color they expected the object to be. From these two pilot tasks, we identified 27 objects that were likely to be known by individuals in the target population (i.e., US students). We also learned the mean expected color of each object, along with the standard deviation around those expected colors (see Persaud et al., 2014, 2021, for a similar approach; means and standard deviations can be found in supplementary materials on OSF). We used this information to form color–object pairings and assess the impact of color–object congruency on memory.^1^

In the current memory task, the expected colors varied in hue only, while saturation and luminance were held constant at 100 and 50 units, respectively. HSL (hue, saturation, and luminance) values were then converted into RGB values to be presented with the objects in MATLAB via a MATLAB color conversion algorithm (Bychkovsky, 2020). Using a Latin square design, across participants, all objects that had well-known color associations were studied with their (1) expected mean color (e.g., a prototypically yellow banana) creating a “strong congruent match,” (2) a color 2 standard deviations away from the expected color, but within the same color category (e.g., an orangish-yellow banana) creating a “weak congruent match,” and (3) a color outside of the expected color category (e.g., purple banana) creating a “strong incongruent mismatch.” Expectation incongruent colors were never near neighbors to the expected category color (e.g., incongruent colors for prototypically yellow objects were never orange or green). Also, different incongruent colors were paired with objects from the same expected color category (e.g., Kermit, broccoli, and Grinch all belong to the green category and their incongruent colors were orange, purple, and red, respectively). Objects with no expected colors were either matched to these colors or paired with a filler color. This design resulted in three study sets of 27 objects. Thus, a third of the participants were shown each study set containing six strong congruent, six weak congruent, six strong incongruent, and nine no congruence object–color pairs. Using a within-subjects design, participants made color recall judgments for both object types (objects with and without prototypical color associations) and across all object–color pairings (congruent matches, weak congruent matches, and strong incongruent mismatches).

**Fig. 1:**
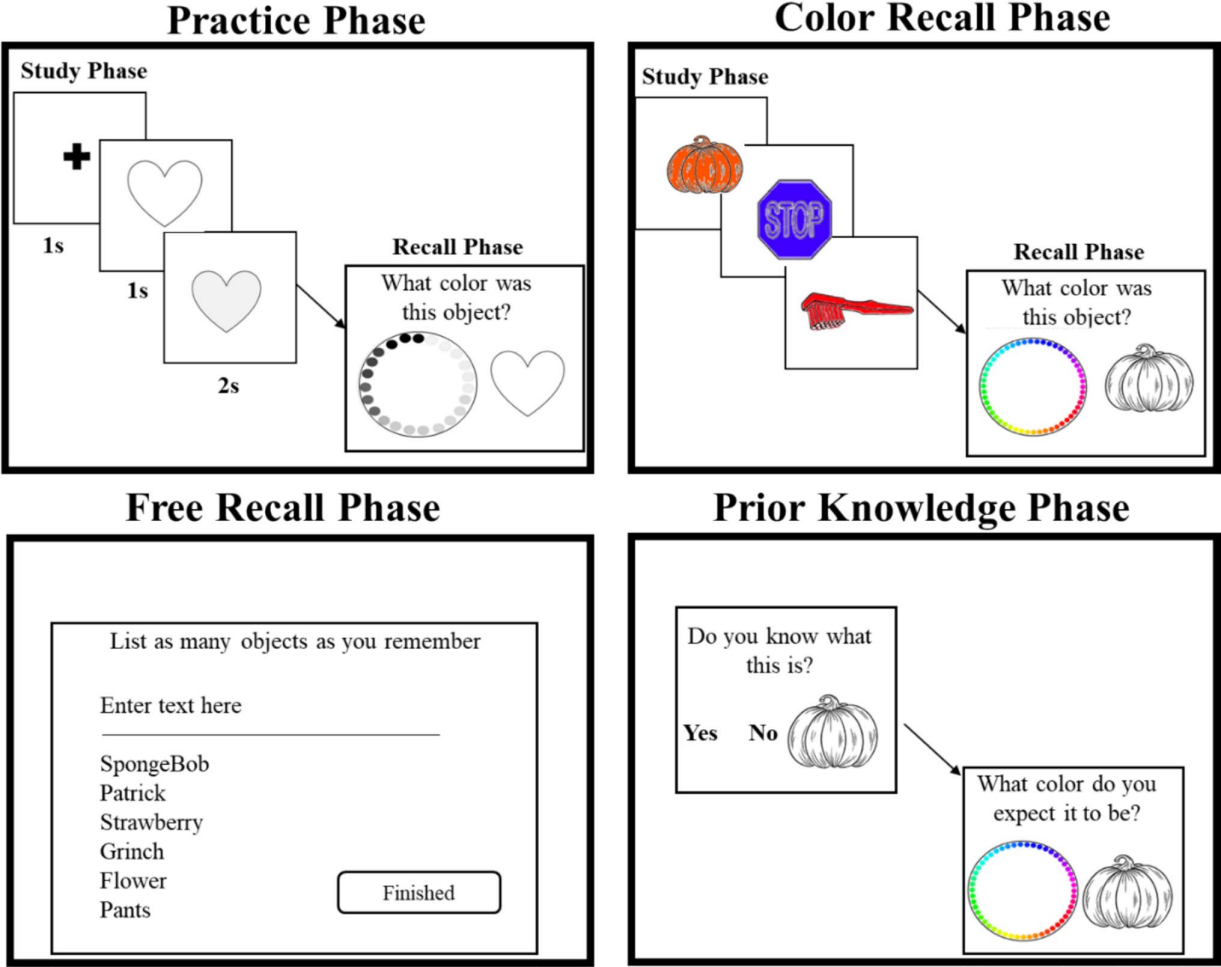
The procedural order of the color recall and object free recall tasks used in Study 1. (Color figure online)

Recall color responses were generated from a color wheel. The color wheel was identical to that used in Persaud et al. (2021), which tested children's and adults’ memory of color. The color wheel sampled colors from the winHSL240 color space. The colors on the color wheel also varied in hue only from 0 to 239, with saturation and luminance held constant at 100 and 50 units, respectively. The color wheel was covered with a white mask that had roughly 50 (¼ inch) holes around its circumference (for a visual of the color wheel, see Fig. 1). Importantly, the holes on the wheel were placed such that the correct target colors could be selected, which would result in zero recall error (i.e., no difference between the color studied and the color recalled). All stimuli were presented on a 15-in. Apple Macintosh display monitor with a vertical refresh rate of 60 Hz. The display was calibrated using X-Rite i1 Pro2 color calibration software. The stimuli were created and administered in MATLAB (Version R2019b, The MathWorks, Natick, MA, USA).

#### Procedure

Participants were told that they would be shown objects of different colors and that they needed to remember the color of each object. Participants were first given two practice trials to familiarize themselves with the task. During practice, participants were shown two shapes (i.e., a heart and a star) one at a time. Each shape was shown for 1 s as a line drawing before filling it with a gray-scaled color (dark gray heart and light gray star) for 2 s, for a total study time of 3 s. The practice trials were conducted in grayscale to familiarize participants with the task but not create interference with the actual colors that would be used in the main study. Participants were then presented with a grayscale color wheel and asked to recall the color that went with each object. All participants identified colors from within the correct color category they studied in the practice trials (e.g., dark-gray colors for heart and light-gray colors for star), indicating their understanding of the task and how the color wheel worked. After the two practice trials, participants were informed that the task would proceed in a similar manner.

The experimental trials immediately followed the practice trials. Participants studied and then recalled the color of unique objects presented in two blocks. Each block consisted of nine objects, followed by the recall task. There were three blocks, totaling 27 uniquely colored objects. In each 9-object block, participants saw two object–color pairings that were strongly congruent, two that were weakly congruent, two that were strongly incongruent, and three that had no congruence. Items were presented one at a time, in randomized order. After nine items were presented, participants were tested by being cued with the line drawing of the object (i.e., void of color) and asked to recall the studied color of the object by clicking on the appropriate location on the color wheel to select the color. The color wheel randomly rotated 45° on each trial. The object presentation order was identical during study and test.

Following the three study–test blocks of object color, participants were then asked to freely recall all the objects that were studied. Responses were typed into a text box presented at the top-center of the screen. Once entered, typed responses were shown below the text box and remained visible until participants were satisfied that they recalled all the objects they could remember. Finally, after free recall, participants were shown each of the 27 objects again, one at a time, and were asked if they knew what the object was (yes or no) and what color they expected the object to be in the real world. They generated expected colors using the color wheel. All responses (color recall, free recall of object names, and color–object expectations) were self-paced. On average the entire study took under 30 min.

### Analysis plan

The study design, hypothesis, analysis plan, and exclusion criteria were preregistered on OSF registries (https://osf.io/szk7f). To evaluate the influence of object–color congruency on episodic memory, we assessed three main dependent variables: the proportion of responses that belonged to the correct color category (e.g., responding with any yellow hue after studying a yellow object), color recall error (the amount of hue shift between study and recalled response), and free recall of objects (whether items were recalled at all). To calculate the proportion of correct category responses, we first classified all responses across each of the congruence conditions into two response types: either within the correct studied color category or not. Given that color category boundaries are subjective and might produce significant individual variation, we used a liberal criterion to decide whether a response came from the study category. Specifically, responses that fell within the means of the neighboring color categories of the target color were classified as within category, while responses that fell outside of the neighboring means were classified as outside of the category. For example, the orange and green color categories border the yellow color category on either side. For recall responses of yellow hue values, responses that were greater than the mean of the orange category, but less than the mean of the green category were classified as falling within the yellow color category. All other responses were considered outside of the target yellow category. Although this is a liberal classification criterion, it avoids strict assumptions about color category boundaries. Color recall error was defined as the difference between recalled hue values and studied hue values.

All memory measures were evaluated for normality and sphericity and were tested with one-way analyses of variance (ANOVAs). For measures that appear to strongly deviate from normality, the data were log-transformed before analyzed. For data that violated sphericity, we report Huynh–Feldt corrected *F* statistic (Girden, 1992). All analyses were conducted in R (R Core Team, 2023). Additional analyses can be found in the online supplementary materials on OSF.

#### Preregistered exclusion criteria

Prior to analysis, we implemented two preregistered exclusion criteria. First, given that the goal of the study was to assess the impact of a priori color–object expectations on feature memory, we removed responses where participants indicated that they did not know the object (~ 2%). Second, for the object–color recall analysis, we sought to analyze the data in two ways. For the first way, we simply examined all data (minus unfamiliar objects as noted in exclusion criteria above); for the second way, we looked to exclude data that could be attributed to random guessing. For this second exclusion criteria, we removed responses that fell outside the means of the neighboring categories (~ 21%). This is a very liberal exclusion criterion which required that responses at least be near the true studied color category to be included. These data were reported as the “within category” analyses. All other recall analyses were performed without this second exclusion. Finally, three additional data points were discarded due to technical error. This resulted in a total of 2% of data removed from the main analysis, and 23% of the data removed for the within category analyses.

### Results

#### Proportion of correct within category responses

**Fig. 2:**
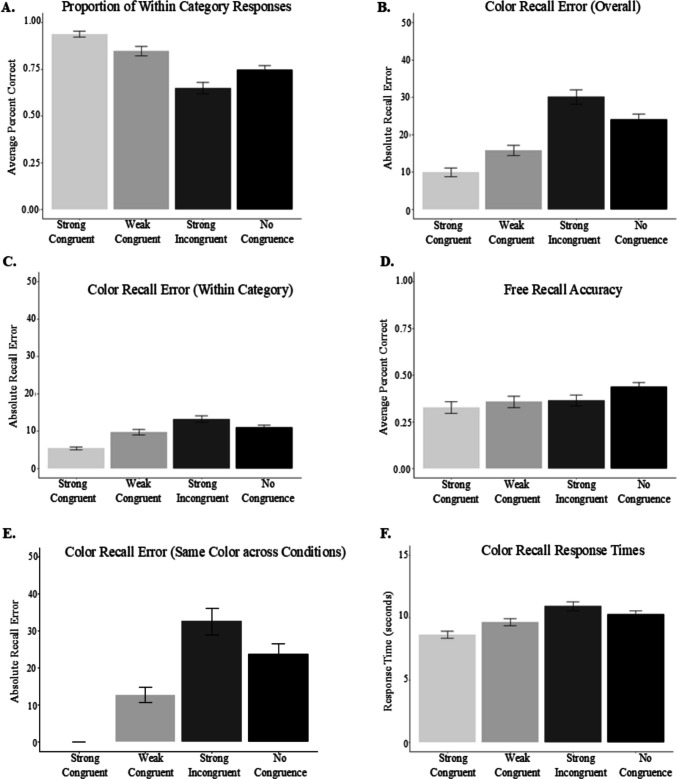
Memory and response time performance for color recall and object free recall tasks. *Note.* Panel **A**. shows the proportion of responses that fell within the target color category. Panels **B.** and **C.** show recall error overall and for responses that fell within the correct category, respectively. Panel **D.** shows free recall performance across conditions. Panel **E** shows differences in error when the same color appeared in different congruence conditions. Panel **F** shows response time performance across congruence conditions. Across all graphs, participants showed the greatest memory error for strongly incongruent color–object pairs. Error bars reflect standard error

To evaluate whether the proportion of color recall responses that fell within the correct color category differed for the strongly congruent, weakly congruent, incongruent, and no congruence object–color pairs, we conducted a one-way repeated-measures ANOVA (Fig. 2A). The results revealed a significant difference in the number of responses that fell within the correct category as a function of congruence condition, *F*(2.64, 118.89) = 0.88 *p* < 0.001, η_p_^2^ = 0.41. Post hoc comparisons (using the Tukey correction to adjust *p*) indicated that strongly congruent items (*M* = 0.94) produced significantly more within category responses than weakly congruent items (*M* = 0.85), *t*(138) = 3.03 *p* = 0.02, strongly incongruent items (*M* = 0.65), *t*(138) = 9.21, *p* < 0.001, and no congruence items (*M* = 0.75), *t*(138) = 6.13, *p* < 0.001. Weakly congruent items produced more within category responses than strongly incongruent items, *t*(138) = 6.25, *p* < 0.001, and no congruence items, *t*(138) = 3.11, *p* = 0.01. In contrast with SLIMM predictions, strongly incongruent items produced significantly less within category responses than no congruence items *t*(138) = − 3.11, *p* = 0.012. Thus, we did not observe the U-shaped curve, particularly in the recall of object colors. Instead, we observed enhanced memory for strongly congruent and weakly congruent items and impaired memory for strongly incongruent items compared with no congruence. Although this finding contrasts with one of the SLIMM predictions, it is supported by another prediction from the model, which suggests that the way we probe memory can influence how expectation-related information is remembered.

#### Overall object–color recall error

Next, we evaluated whether recall error (difference between recalled hue values minus studied values) differed as a function of congruence condition (Fig. 2B). A repeated-measures ANOVA showed a significant difference in log recall error for the color objects across the strongly congruent, weakly congruent, strongly incongruent, and no congruence color–object pairs, *F*(3, 141) = 60.38, *p* < 0.001, η_p_^2^ = 0.56. Post hoc comparisons (using Tukey adjusted *p*) indicated that strongly congruent items (*M* = 9.96) produced significantly less recall error than weakly congruent items (*M* = 15.79), *t*(141) = − 5.83, *p* < 0.0001, strongly incongruent items (*M* = 30.09), *t*(141) = − 12.61, *p* < 0.0001, and no congruence items (*M* = 24.06), *t*(141) = − 10.37, *p* < 0.001. Weakly congruent items produced significantly less error than strong incongruent items, *t*(141) = − 6.84, *p* < 0.0001, and no congruence items, *t*(141) = − 4.00, *p* = 0.0006. As with the within category analysis, strongly incongruent items produced significantly more recall error than no congruence matches, *t*(141) = 3.54, *p* = 0.003.

#### Object–color recall error (within category)

Because the same recall error pattern could be produced if participants simply guessed and used only prior knowledge to generate responses, we also evaluated whether recall error significantly differed for responses that fell within the correct color category (Fig. 2C). A repeated-measures ANOVA showed a significant effect of congruence condition on recall error, *F*(2.01, 94.47) = 0.67, *p* < 0.0001, η_p_^2^ = 0.33. Post hoc comparisons (using Tukey adjusted *p*) indicated that strong congruent items (*M* = 5.42) produced significantly less recall error than weak congruent items (*M* = 9.60), *t*(141) = − 4.37, *p* = 0.0001, strong incongruent items (*M* = 13.15), *t*(141) = − 7.74, *p* < 0.0001, and no congruence items (*M* = 10.92), *t*(141) = − 6.20, *p* < 0.0001. Weak congruent items produced significantly less error than strong incongruent items, *t*(141) = − 3.64, *p* = 0.002, but no difference from no congruence items, *t*(141) = − 1.47 *p* = 0.46. Strong incongruent items produced marginal (adjusting for multiple comparisons), but not significantly more error than no congruence matches, *t*(141) = 2.52, *p* = 0.06.

#### Object free recall

Finally, we evaluated free recall for the objects, as a function of the congruence conditions (Fig. 2D). Means were not very high (39%) and also not different across conditions (strong congruent: 35%, weak congruent: 39%, strong incongruent: 39%, no congruence: 44%). A repeated-measures ANOVA revealed no significant difference in the number of objects freely recalled based on congruence condition, *F*(3, 184) = 0.42, *p* = 0.74.

#### Additional exploratory analyses

##### Recall error for same color values across congruence conditions

Past research suggests that there are certain colors in general, independent of objects, that are more accurately recalled than others (Bae et al., 2015; Persaud & Hemmer, 2016; Persaud et al., 2021). To examine whether the colors presented with congruent versus incongruent objects were indeed better recalled, we compared memory when the same color hue values were paired with congruent objects (e.g., orangish-red pumpkin), incongruent objects (e.g., orangish-red broccoli), or no congruence objects (e.g., orangish-red toothbrush). Given that it is the same exact color, any differences in recall error can be attributed to the color’s congruence relationship with the paired object. Since the same color hue could not be used for both the strong congruent and the weak congruent conditions, here we only evaluate error for weak congruent items, strong incongruent items, and no congruence items. There was a total of seven such colors. A one-way repeated-measures ANOVA revealed a significant difference in log recall error for the same color hue values when studied as weak congruent items, strong incongruent items, and no congruence items, *F*(1.81, 84.88) = 0.90, *p* = 0.0001, η_p_^2^ = 0.22. Post hoc comparisons (using the Tukey method for adjusted *p*) indicated that the same color hue values were recalled with less error when studied as weak congruent items (*M* = 12.78) compared with strong incongruent items (*M* = 32.50), *t*(94) = − 5.14, *p* < 0.0001, and no congruence items (*M* = 23.69), *t*(141) = − 3.40 *p* = 0.003. There was no significant difference in recall error for the same hue values as strong incongruent and no congruence matches, *t*(94) = 2.09, *p* = 0.10. Interestingly, when we look at only responses that fell within the correct color category, this difference in log recall error for the same color hue values across congruence conditions became marginal, but not significantly different, *F*(2, 4.94) = 12.03, *p* = 0.085.

##### Response time across congruence conditions

 We also explored whether response times differed across the congruence conditions (Fig. 2F). A repeated-measures ANOVA revealed a significant difference in response time during color recall as a function of congruence condition, *F*(3, 141) = 22.07, *p* < 0.0001, η_p_^2^ = 0.32. Post hoc comparisons (using the Tukey adjusted *p*) indicated that recall responses were significantly faster for strong congruent items (*M* = 8.18 s) compared with weak congruent items (*M* = 9.25 s), *t*(141) = − 2.71, *p* = 0.04, strong incongruent items (*M* = 10.40 s), *t*(141) = − 6.46 *p* < 0.0001, and no congruence items (*M* = 9.64 s), *t*(141) = − 4.88, *p* < 0.0001. Recall responses were also faster for weak congruent items compared with strong incongruent items, *t*(141) = − 3.78, *p* = 0.001. There were no significant differences between any other pairwise comparisons.

### Discussion

Contrary to our initial prediction, we did not find a U-shaped pattern in object feature recall of expectation related items. Instead, we found that recall was only better for object features that were highly congruent with people’s prior expectations compared with no congruence items. In color recall, congruent items appeared to improve accuracy by helping to stay within the correct studied category, reducing the amount of memory search time needed to retrieve the studied feature (evidenced by response time), and reducing overall feature error. In contrast, incongruent items led to more out of category responses, more memory search time needed for retrieval, and overall, more error. Incongruent items led to worse memory, even compared with items that did not have strong expectations.

The poorer memory performance for incongruent items might be a function of probing memory using recall as opposed to recognition (Sherman & Frost, 2000; van Kesteren et al., 2013). Recall is thought to be facilitated by memory search processes that are better at retrieving expectation-congruent information as opposed to expectation-incongruent information. Thus, incongruent item features might be sufficiently encoded and stored, but difficult to retrieve in recall. In recognition memory, which eliminates the need to employ memory search processes, the deficit in performance for incongruent items might be attenuated. Thus, the goal of Study 2 was to test recognition memory for object–color pairs.

## Study 2: Recognition of object–color pairs

In Study 2, we examined individuals’ recognition memory for congruent, incongruent, and no congruence item features. We measured recognition memory using a four-alternative forced-choice (4 AFC) task in which adults had to identify the object–color pairs they had previously studied.^2^ In study 2, we only included weakly congruent items in the congruent condition, and made the strong congruent items from Study 1, one of the four recognition choices. This ensured that accuracy in the congruent condition would not be solely based on adults’ strategic guessing with prior knowledge as this strategy would result in an inaccurate response.

The experimental procedure used in Study 2 was akin to that of Study 1 in that, in addition to the main memory task, participants also completed a free recall task for object names and the prior knowledge assessment (Study 1). Although Study 1 revealed no significant differences in the free recall of the object labels based on congruence conditions, past studies have found differences in the free recall of objects with congruent and incongruent color features (Morrita & Kambara, 2022). Therefore, we included the free recall task in Study 2 to ensure continuity across Studies 1 and 2 and determine whether this lack of a difference in free recall would replicate, given the mixed findings in the literature.

While the experimental procedures for Study 1 and 2 were relatively similar, there were a few differences. First, anticipating that the recognition task would be easier (e.g., Freund et al., 1969), we removed the blocked design and presented participants with all 27 objects in succession. Second, as mentioned earlier, for objects that had well-known color associations, we removed the strong congruent condition and only included the weak congruent condition. Finally, given the residual impacts of the pandemic, recruitment of in-person participants proved challenging. Therefore, we transitioned to an online paradigm, allowing us to reach our target sample size.

We predicted that while recall revealed better memory for congruent over incongruent and no congruence item features as in Study 1, recognition memory would reveal better memory for congruent *and* incongruent item features relative to no congruence item features.

### Methods

#### Participants

Forty-five undergraduate students at Rutgers University–Newark participated in this study for course credit as compensation. All participants provided self-reports of normal or corrected-to-normal vision and color vision. All participants were run online via the Zoom video conference software and gave written informed consent prior to the start of the study. Previous research has found a medium-sized effect of incongruence on memory (*d* = 0.57; Greve et al., 2017) when testing 20 subjects. Based on this medium effect size and our current design, we planned to recruit a total of 30 nonexcluded participants to test for the main effect of incongruence on memory. Four participants were dropped due to technological or experimental error. One participant was dropped due to failure to complete the study. The final sample consisted of 40 participants which provided 95% power to detect a medium incongruency effect. At the end of the study, participants were invited to ask the experimenter any questions related to the study. This study was approved by the Rutgers University–Newark Institutional Review Board.

#### Stimuli

The stimuli were similar to those in Study 1, except for the exclusion of the strong congruent condition. The stimuli consisted of 27 objects— nine congruent,^3^ nine incongruent, and nine objects not associated with strong color expectations. All objects that had well-known color associations were studied with either (1) a color two standard deviations away from the expected color but within the same color category (e.g., dark-orange pumpkin) creating a “congruent match” or (2) a color outside of the expected color category (e.g., green pumpkin) creating an “incongruent mismatch.” The object–color pairs for objects with well-known color associations were counterbalanced, such that half the participants studied the objects with a congruent color, and the other half studied those objects paired with incongruent colors. Objects with no strong color associations (e.g., toothbrush) were either matched to these colors or paired with an alternative filler color.

**Fig. 3:**
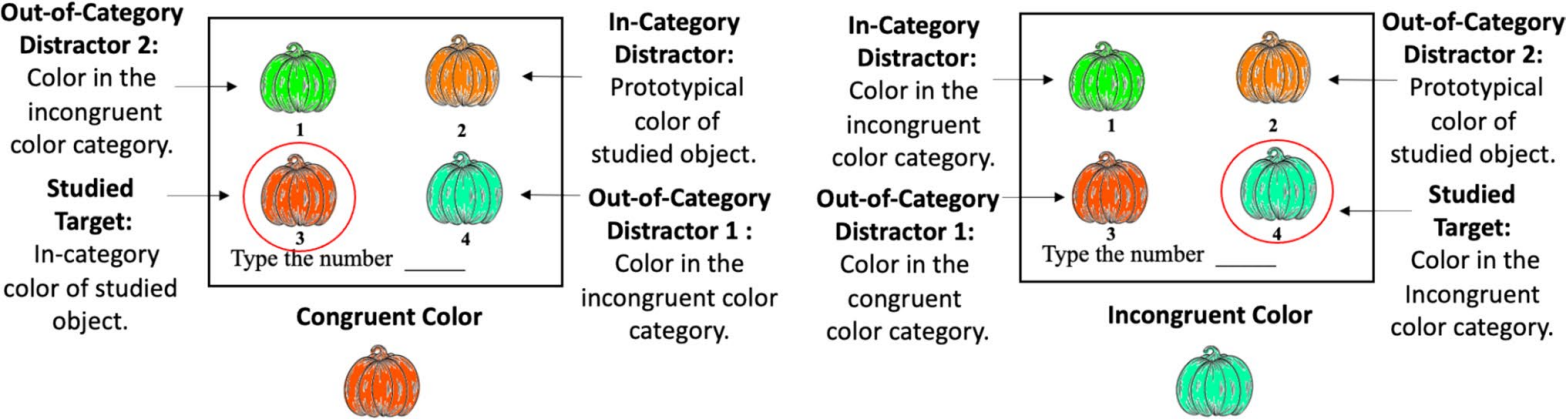
4 AFC response options for items in the congruent and incongruent color conditions. (Color figure online)

There were four options participants could select from during the recognition task: the studied target and three distractors (see Fig. 3). The studied target was the object–color pair participants initially studied. The three distractors were created using the same color expectations learned from the initial prior knowledge pilot study. For the congruent condition (Fig. 3, left panel), one of the distractors was the true expected color for the object (i.e., in-category distractor). The in-category distractor was equivalent to the strong congruent condition from Study 1. The remaining two distractors were two colors sampled from a non-neighboring color category (i.e., out-of-category distractor 1 & 2). For example, for pumpkin, the true expected color category was orange, and a non-neighboring color category was green. For the incongruent condition (Fig. 3, right panel), one of the distractors came from the same color category as the target color (i.e., in-category distractor). The remaining two distractors came from a non-neighboring color category. Importantly, the non-neighboring color category in the incongruent condition was also the true expected color category for the object (e.g., orange when pumpkin was the object). As such, one of the out-of-category distractors was the true expected color for the object (i.e., out-of-category distractor 2) and the second out of category distractor was another color sampled from that color category. All stimuli were presented on a 15-in. Apple Macintosh display monitor with a vertical refresh rate of 60 Hz. The stimuli were created and administered in MATLAB (Version, R2019b) online via Zoom’s screen-sharing feature.

#### Procedure

The experimental task was conducted in four phases: a practice phase, a recognition phase, a free recall of objects phase, and a prior knowledge assessment phase. Using Zoom’s remote-control feature, participants indicated their recognition and free-recall responses by controlling the shared screen and typing the number corresponding to each remembered item recognition or the label of a studied object.

##### Practice phase

 In the practice phase, participants completed two trials where they studied and recognized the color of two grayscale shapes—a heart (dark gray) and then a star (light gray). Each shape was preceded by a 1-s fixation cross, followed by a line drawing of the shape for 1 s and then a filled-in grayscale shape for 2 s, for a total study time of 3 s. Participants were then presented with a 4 AFC question for each studied object. Participants identified which color went with each shape (e.g., dark-gray heart) by typing the number that corresponded with their answer choice in a text box located at the bottom of their screen (see Fig. 3). All participants accurately identified the colors studied in the practice trials, indicating an understanding of the experimental task.

##### Recognition phase

**Fig. 4:**
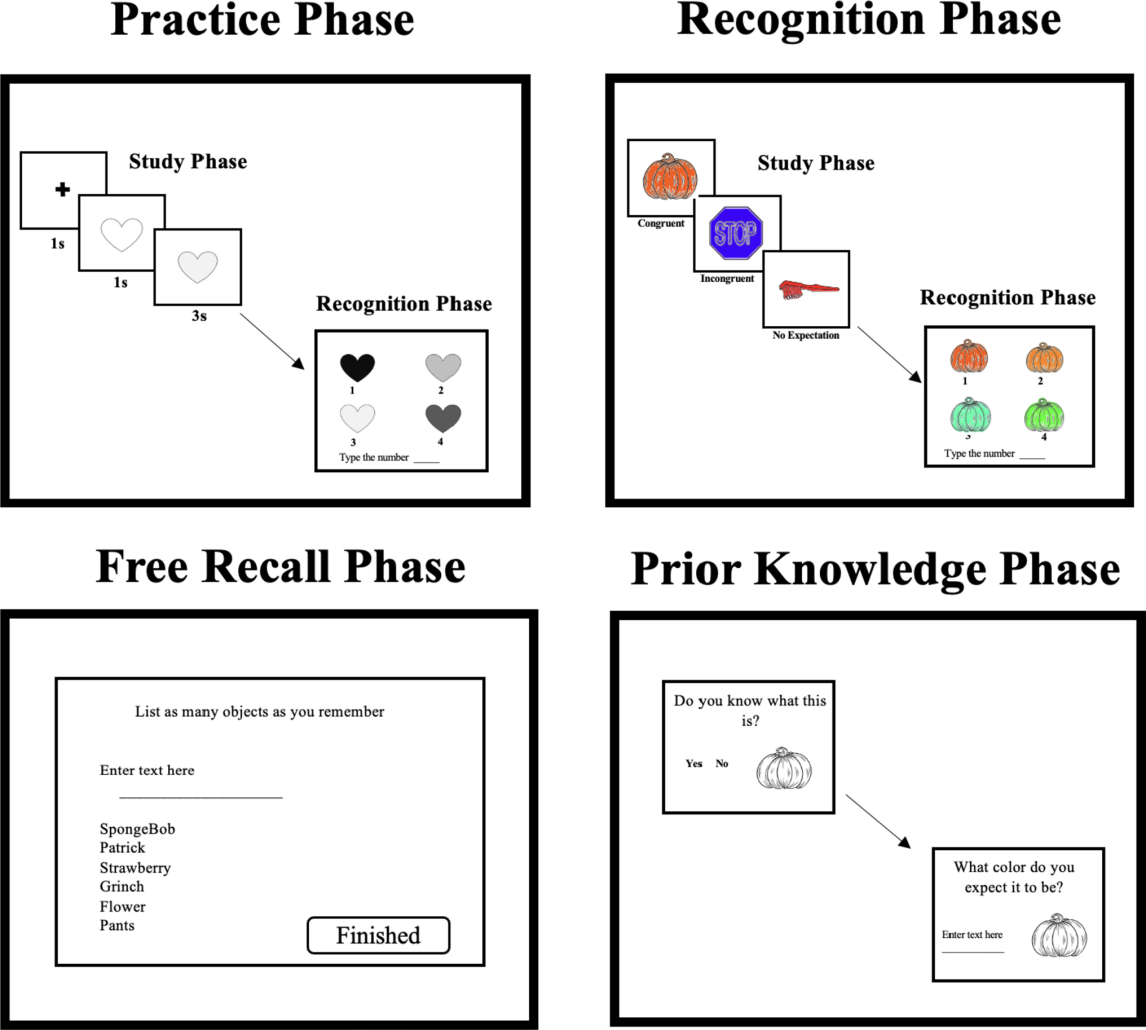
The procedural order of the color recognition and free recall tasks used in Study 2. (Color figure online)

Immediately following the practice trials, participants moved on to the main experimental task where they studied and recognized 27 unique object–color pairs that fell into the three color congruence conditions (e.g., congruent, incongruent, no congruence). The objects were presented consecutively in a randomized order. Identical to the practice trials, each object–color pair was preceded by a 1-s fixation cross and then shown as a line drawing for 1 s and, finally, as a filled-in object for 2 s, for a total study time of 3 s. Participants studied each object–color pair one at a time. At test, participants made color recognition judgments for all studied object–color pairings. In a 4-AFC design, participants identified studied object–color pairs by typing in the number that corresponded with their answer choice in a text box located at the bottom right of the screen (see Fig. 4).

##### Recall phase

Similar to Study 1, we also explored whether the congruence of object–color pairs impacts memory for objects in general. In a free recall task, participants were asked to type as many object names as they could remember. Responses were typed into a text box presented at the top center of the screen. Once entered, typed responses were shown below the text box and remained visible until participants were satisfied that they recalled all the objects they could remember. Responses were self-paced.

##### Prior knowledge assessment

Afterward, participants completed a prior knowledge assessment in which they were shown line-drawings of each of the 27 studied objects again, one at a time, and were asked two questions: “Do you know what this is?” (yes or no) and “What color do you expect this object to be?” Participants indicated their expected colors by typing the color label into a text box located to the center left of the screen (see Fig. 4). All responses were self-paced.

### Analysis plan

The study design, hypothesis, analysis plan, and exclusion criteria were preregistered on As Predicted (https://aspredicted.org/sx7q-2cqg.pdf). Following our preregistered exclusion criteria, we removed responses where participants indicated that they did not know what the object was (~ 0.02%). To analyze memory performance, we first calculated recognition accuracy for each participant as the number of correctly identified color–object pairs divided by the total number of objects studied. Next, we calculated free recall accuracy as the number of correctly recalled objects divided by the total number of objects studied. To assess whether recognition and recall accuracy varied across congruence conditions (i.e., congruent, incongruent, and no congruence), we conducted two separate one-way repeated-measures ANOVAs. To assess whether there were differences in response time based on congruence condition, we compared the average response time for items in each of the congruence conditions for both recognition and recall. We also performed several exploratory analyses to further evaluate the interactions between congruence and recognition memory. All analyses were conducted using R (R Core Team, 2023).

### Results

#### Color recognition accuracy 

**Fig. 5:**
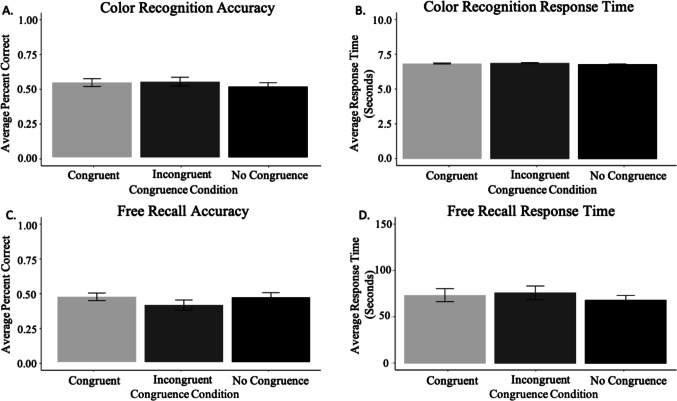
Memory performance and response time for color recognition and free recall tasks. *Note.* Panel **A.** shows the mean color recognition accuracy (proportion correct) in Study 2. Panel **B.** presents the average response time for the color recognition task in Study 2. Panel **C.** illustrates the mean object Free Recall accuracy in Study 2. Panel** D.** displays the average response time for the object free recall task in Study 2. Error bars reflect standard error

We evaluated recognition accuracy, as a function of the color congruence conditions. In general, participants performed above chance (congruent mean: 52%, binomial *p* < 0.0001; incongruent mean: 53%, binomial, *p* < 0.0001; no congruent mean: 49%, binomial p < 0.0001). A repeated-measures ANOVA revealed no significant difference in recognition accuracy of object–color pairs based on congruence condition, *F*(2, 114) = 1.54, *p* = 0.219 (see Table 1 and Fig. 5A).

**Table 1:** Average memory performance across memory tasks and color conditions

Memory task	Congruence conditions		
	Congruent	Incongruent	No congruence
Recognition memory	0.52 (0.03 *SE*)	0.53 (0.03 *SE*)	0.49 (0.03 *SE*)
Free recall memory	0.46 (0.03 *SE*)	0.40 (0.04 *SE*)	0.45 (0.03 *SE*)

#### Object free recall

Next, we evaluated free recall for the objects, as a function of the congruence conditions. In general, participants recalled about half of the items (congruent: 46%; incongruent: 40%; no congruence: 45%). Similar to the results of Study 1, a repeated-measures ANOVA revealed no significant difference in the number of objects freely recalled based on congruence condition, *F*(2, 112) = 0.016, *p* = 0.985 (see Table 1 and Fig. 5C).

#### Additional exploratory analysis

##### Response time

We conducted an exploratory analysis to probe whether response times differed across the three congruence conditions in the color recognition and object-free recall memory tasks. Response time differences may indicate how difficult it was for participants to recognize or recall items across different congruence conditions, with longer response times potentially reflecting greater difficulty. For both color recognition and free recall, response time was measured as the time between the start of the trial and when participants submitted a response (either a recognition choice or a typed response). A repeated-measures ANOVA revealed no significant difference in recognition response time as a function of congruence condition, *F*(2, 11) = 1.22, *p* = 0.89 (Fig. 5B). Similarly, we found no statistically significant difference in free recall response time as a function of congruence condition, *F*(2, 112) = 1.05, *p* = 0.35 (Fig. 5D). These findings suggest that in both the recognition and free recall tasks, participants took relatively similar lengths of time to respond across congruence conditions (see Table 2).

**Table 2:** Average response time across memory tasks and congruence conditions

Memory task	Congruence conditions		
	Congruent	Incongruent	No congruence
Recognition memory	6.67 s (0.03*SE*)	6.70 s (0.03 *SE*)	6.62 s (0.03 *SE*)
Free recall memory	69.54 s (6.65 *SE*)	72.02 s (6.96 *SE*)	64.55 s (4.69 *SE*)

##### Types of recognition error

We conducted additional exploratory analyses to evaluate how expectation congruence might shape the kinds of errors participants made in their recognition judgments. The current research design allowed us to assess whether individuals may have encoded the general color category or the specific color of an item by observing the errors they made and whether they vary based on the congruence condition.

In the congruent condition, if individuals encode the specific color of an item, we should expect them to accurately recognize the studied object color at test. However, if individuals encode the general color category of an item, we might expect them to make an incorrect recognition response within the same color category as the target color (i.e., in-category distractor). If individuals misremembered both the specific item color and the general item color category, they would still likely choose an incorrect color within the same color category as the target color, as this option is consistent with their prior knowledge, producing a “good guess” of the studied color.

The same is true in the incongruent condition. If individuals encode the specific color or the general color category, they would either accurately recognize the studied item or choose the incorrect color within the same color category as the target color (i.e., in-category distractor). However, if individuals misremembered the target color and the general color category, they are more likely to select an outside category distractor option, specifically the true expected color for the object (i.e., out-of- category distractor 2). This would equate to guessing based on prior knowledge.

In the no congruence condition, if individuals encode the specific color we would expect them to accurately recognize the studied object at test. If they encode the general color category of a studied item, like in the other congruence conditions, we might expect them to make an incorrect choice within the same color category as the target color (i.e., in-category distractor). However, if individuals “lose” both the specific item color and the general item color category, they would be equally as likely to pick any option (choose randomly), as they have no prior knowledge for the studied item that would pull them toward an expected color.

Therefore, if it is the case that participants have at least somewhat stronger memory traces for both the congruent and incongruent objects compared with the no congruence objects, even when they do not choose the correct target option, we might expect a larger proportion of incorrect recognition responses to come from the same color category as the target color (i.e., in-category distractor) compared with the two outside category distractor options (i.e., out-of-category distractors 1 & 2). However, if individuals misremember object–color associations in the congruent and incongruent conditions, individuals should guess with their prior color expectation. Guessing with an a priori color expectation in the congruent condition would also lead to a higher proportion of within-category distractor responses. In contrast, the incongruent condition would lead to a higher proportion of out-of-category distractor (i.e., expected color) responses (i.e., out-of-category distractor 2). For the no congruence condition, when individuals incorrectly recognize a studied object, they should do so randomly, choosing both in-category and out-of-category distractor responses equally.

We conducted a 3 (congruent, incongruent, no congruence) × 3 (in-category distractor, out-of-category distractor 1, out-of-category distractor 2) repeated-measures ANOVA to investigate whether congruence condition and response type influenced the proportion of incorrect responses chosen. There was a statistically significant main effect of response type, *F*(2, 342) = 14.05, *p* < 0.001, η_p_^2^ = 0.08, but no main effect of congruence condition, *F*(2, 342) = 1.06, *p* = 0.35. There was also a marginal but nonsignificant interaction between response type and congruence condition, *F*(4, 342) = 2.25, *p* = 0.09. Post hoc comparisons (using Tukey adjusted *p*) revealed that the proportion of in-category distractor responses were significantly greater than both the out-of-category distractor 1, *t*(342) = 4.31 *p* = 0.0001, and out-of-category distractor 2, *t*(342) = 4.83 *p* < 0.0001. There was no significant difference between the proportions of the two out of category distractors, *t*(342) = 0.52 *p* = 0.86.

Despite the lack of a significant difference between the proportion of response types chosen between the congruence conditions, looking at the percentage of the types of errors made overall reveals an interesting pattern (see Table 3). Specifically, congruent items that were misremembered were more likely to be misremembered as within-category distractors (74%). The same is true for no congruence items (54% within category and 46% out of the category). Whereas incongruent items were just as likely to be misremembered as out-of-category distractors (50%) as within-category distractors (50%).

**Table 3:** Average percentage of recognition responses across congruence conditions and response type

Congruence condition	Response type			
	Study target	In-category distractor	Out-of-category 1 distractor	Out-of-category 2 distractor
Congruent	0.52	0.36 (*74%*)	0.07 (*14%*)	0.06 (*12%*)
Incongruent	0.53	0.23 (*50%*)	0.09 (*20%*)	0.14 (*30%*)
No congruence	0.49	0.28 (*54%*)	0.12 (*24%*)	0.11 (*22%*)

In one final analysis of errors, we combined the two out-of-category distractor errors and compared them to the in-category distractor errors. The goal for combining the two out-of-category was two-fold: since we did not predict differences between the out-of-category distractors for two of the three color congruence conditions (i.e., the strong congruent and no congruence conditions) and to potentially increase power for this ad hoc analysis. We conducted a 3 (congruent, incongruent, no congruence) × 2 (in-category distractor, out-of-category distractor) repeated-measures ANOVA to investigate whether congruence condition and error type influenced the proportion of incorrect responses made. There continued to be significant main effect of response type, *F*(1, 348) = 27.96, *p* < 0.0001, η_p_^2^ = 0.07, and no main effect of congruence condition, *F*(2, 348) = 1.06, *p* = 0.35. However, the interaction between response type and congruence condition was statistically significant, *F*(2, 348) = 3.63, *p* = 0.03, η_p_^2^ = 0.02. Post hoc comparisons using Tukey’s HSD revealed a few interesting response patterns. In the congruent condition, participants made significantly more in-category errors than out of category errors, *t*(348) = 4.85*, p* < 0.0001. The same pattern was observed for the no congruence condition, *t*(348) = 3.26*, p* = 0.02. However, there were no differences in errors for the incongruent condition, *t*(348) = 1.05*, p* = 0.90. There were more in-category errors in the congruent condition compared in-category errors in the incongruent condition, *t*(348) = 3.03*, p* = 0.03. These results seem to suggest that participants are sensitive to the congruency of studied information and this sensitivity influences the pattern of errors they make. When they have some memory trace that what they studied was congruent with their prior expectations, they leverage this sensitivity to choose options that are close to the congruent category. However, when the study items are incongruent with strong expectations, they make more diverse errors.^4^

## Discussion

In Study 2, we investigated whether object–color congruence influences recognition memory. Initially, we predicted that participants’ memory for incongruent object–color pairs would be comparable to their memory for congruent object–color pairs. However, we also expected memory for both to be better than memory for no congruence pairs. Contrary to our initial prediction, we did not find a difference in recognition memory across congruence conditions. Similarly, we explored free recall for studied objects and found no difference in memory performance across congruence conditions. Taken together, these findings suggest that all objects, regardless of condition, were equally well encoded and retrieved in both the recognition and free recall tasks.

Additionally, we conducted two exploratory analyses: response time and error response type analyses. While there was no difference in response time, there were differences in the types of response error participants made. When participants made errors, they were significantly more likely to choose an option from the correct target color category rather than from an outside color category. Interestingly, we observed a marginal but nonsignificant interaction between response type and congruence condition. Participants chose the in-category distractor option slightly more often when the object pair was congruent compared with the incongruent and no congruence conditions. When we looked at the percentage of the types of errors made across congruence conditions more closely, an interesting pattern emerged. That is, when congruent items were mis-remembered they were more likely to be mis-remembered as an option from the correct target color category rather than from an outside color category, while incongruent and no congruence recognition errors appear more random. This pattern of recognition error may suggest that participants are using different strategies across congruence conditions, relying on their well-established prior knowledge in the congruent condition and choosing more randomly in incongruent and no congruence conditions. However, since the interaction is nonsignificant, it should be interpreted with caution.

Surprisingly, we did not find better recognition memory for the two congruence conditions relative to the no congruence condition. It is possible that the lack of a difference in recognition memory, across all congruence conditions, signaled an insensitivity to the expectation (in)congruence of the studied object color pairs for the purposes of aiding or influencing memory. Past research suggests that for memory effects to occur, individuals must not only have expectations but also activate those expectations and deem them relevant for learning in the current study context (Brod, 2021). We return to this point in the General Discussion.

Finally, Studies 1 and 2 directly tested memory for object features with strong a priori expectations, such as object color. To delve deeper into the relationship between congruence and memory, and to test a third prediction of the SLIMM model, in Study 3, we examined how congruence might influence memory for a potentially expectation-irrelevant study feature: object shape.

## Study 3: Shape recognition for object–color pairs

The purpose of Study 3 was to evaluate a third prediction of the SLIMM account that expectation-incongruent, but not congruent scenarios may enhance memory for incidental, expectation-irrelevant features of studied events. To test this prediction, we again paired the objects used in Studies 1 and 2 with congruent and incongruent colors and here assessed participants’ recognition memory of the shape of those objects (i.e., an expectation-irrelevant feature^5^). Since this study considered memory for a feature that was unrelated to the expectation (i.e., object shape instead of object color), guessing with prior knowledge could no longer create a disadvantage for any of the color congruence conditions, but especially the incongruent condition. As such, we returned to comparing performance in the strong congruent and incongruent conditions. We also sought to understand whether the strength of the congruence of the expectation-relevant feature impacts memory for the expectation-irrelevant feature. As such, we included the weak congruent condition as well, for a total of three congruence conditions. Further, given that this third prediction only involves congruent and incongruent scenarios, we opted to simplify the design by excluding the no congruence condition. We expected to observe better memory for the expectation-irrelevant feature in the strong incongruent condition compared with the strong congruent and no congruence conditions. Finally, note that, like Study 2, Study 3 was also conducted during the tail end of the COVID-19 pandemic. As such, these data were collected in an unmoderated study ran online.

### Methods

#### Participants

One hundred and thirty-five participants were recruited from the Cloud Research Connect online subject pool (Hartman et al., 2023) to participate in Study 3 for monetary compensation. The study restricted the sample to US participants only. Data from one participant was discarded due to a failure to follow task instructions. Given that Study 3 was unmoderated and collected online, we sought to substantially shorten the task by including a third of the number of trials used in the prior two studies. Thus, the increase in number of participants in Study 3 was to ensure that a similar number of data points for the congruence conditions could be achieved. The final sample yielded 95% power to detect a medium to large effect. All participants reported having normal or corrected-to-normal vision and color vision, and gave informed consent prior to the start of the study. This study was approved by Rutgers University–Newark Institutional Review Board.

#### Stimuli

The stimuli consisted of nine of the objects used in Studies 1 and 2 (four natural objects, four popular cartoon characters, and one man-made object). The objects were chosen on the basis of them having an expected color that spanned the seven basic color categories. Each object was then paired with the strong congruent color, a weak congruent color, and an incongruent color. This design resulted in three study sets of nine objects. A third of the participants were shown each study set. Using a 10-alternative forced choice design, participants made shape recognition judgments for the objects based on the three different color pairings.

The 10 alternatives for the recognition phase consisted of the target object and three different exemplars of the target object. Each of the four objects were then morphed to create either two or three different versions by either changing the width, height, orientation, or a combination of the three. To ensure that participants could not simply guess the target shape object, we conducted a separate pilot study (*N* = 18) where participants were asked to rank order the alternatives in terms of prototypicality. The target object was chosen such that it was never rated as the most prototypical shape for the object.

#### Procedure

**Fig. 6:**
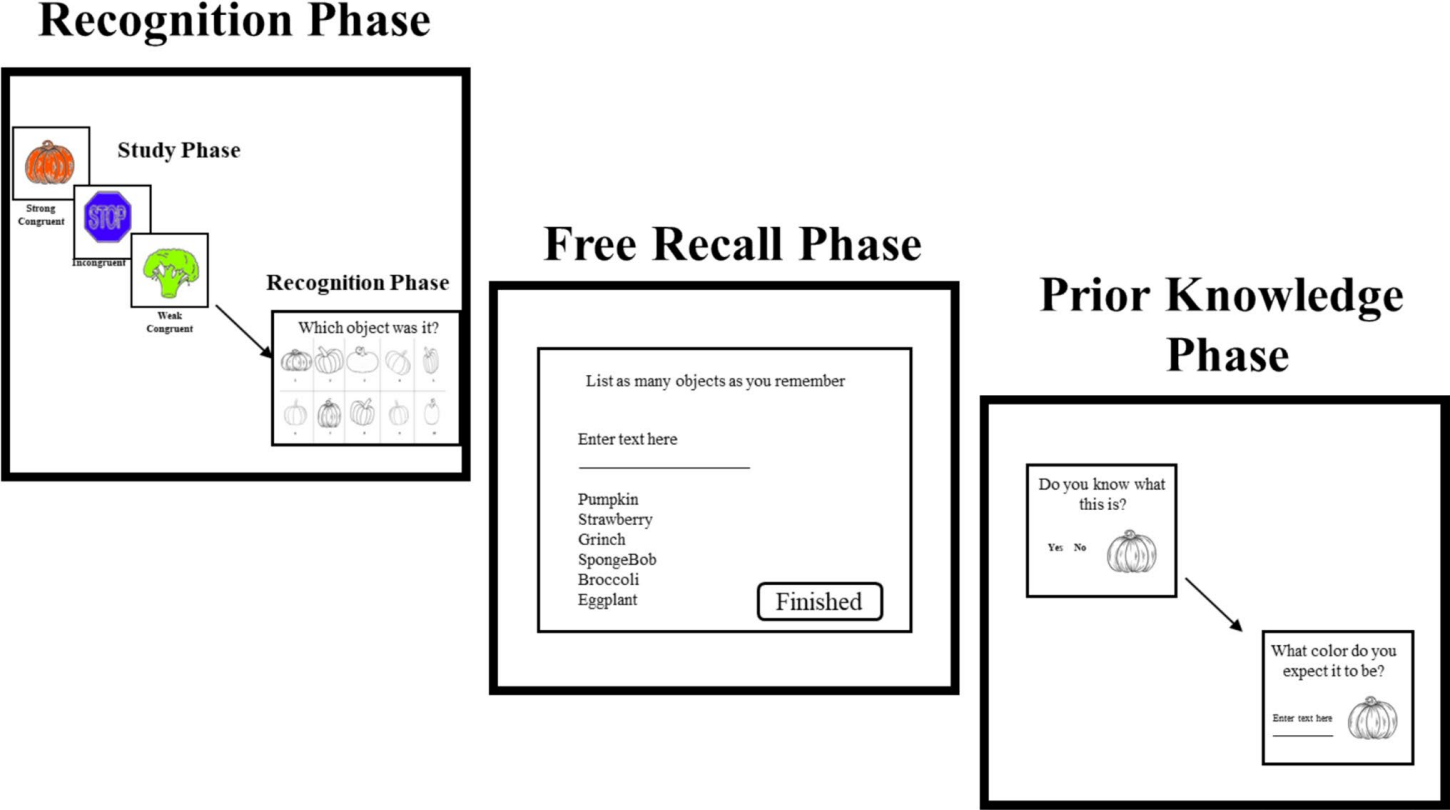
The procedural order of the shape recognition and free recall task used in Study 3. (Color figure online)

The procedure was similar to Study 1 and Study 2. Participants were told that they would be shown different objects and that they needed to remember each object as they would later be tested on their memory for the objects. Each shape was shown for 1 s as a line drawing before it was filled with a color for 2 s, for a total study time of 3 s. Participants were then presented with 10 alternatives of the target object unfilled with color and were asked to recognize the object they remembered studying. Participants studied all nine objects within a single block, one at a time. The object presentation order was identical during study and test. After participants completed a free recall task, where they were asked to name all of the objects they remembered seeing. Following the memory task, participants then saw each object again, one at a time, and were asked if they knew what the object was and what color they expected the object to be. Their judgments of whether they knew the objects determined whether the objects were included in the subsequent analysis (see Fig. 6). All tasks were self-paced.

#### Analysis plan

Similar to Studies 1 and 2, we removed responses where participants indicated that they did not know what the object was (~ 0.04%). We calculated shape recognition accuracy for each participant as the number of correctly identified shapes divided by the total number of objects studied. Next, we calculated free recall accuracy as the number of correctly recalled objects divided by the total number of objects studied. We then employed one-way repeated-measures ANOVAs to evaluate whether color congruence impacts shape recognition, free recall, and response time. All analyses were conducted using R (R Core Team, 2023).

### Results

#### Proportion correct recognition

**Fig. 7:**
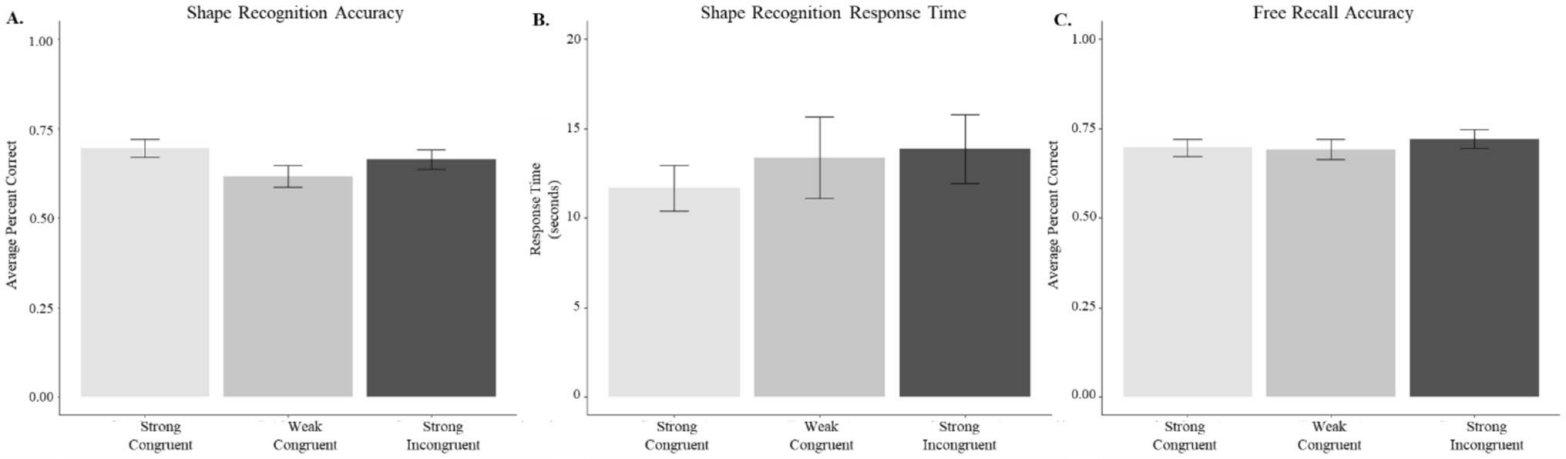
Memory accuracy and response time performance for shape recognition task. *Note.* Panel **A.** shows the proportion of correct shape recognition across congruence conditions. Panel **B.** shows response time, and Panel **C.** shows free recall of objects across conditions. Error bars reflect standard error

To evaluate whether recognition accuracy for the shape of objects differed as a function of congruence condition, we conducted a one-way repeated-measures ANOVA (Fig. 7A). The results revealed a significant difference in recognition accuracy of object shape between the strong congruent (*M* = 0.69), weak congruent (*M* = 0.62), and strong incongruent (*M* = 0.66), *F*(2, 266) = 3.09, *p* = 0.047, η_p_^2^ = 0.02. Post hoc comparisons (using Tukey adjusted *p*) indicated that the shape of objects paired with congruent colors were better remembered than object shapes paired with weak congruent colors, *t*(266) = 2.47 *p* = 0.038, but no other pairwise comparisons were significant.

#### Response time

We also explored whether response times during shape recognition differed across the object–color pairs (Fig. 7B). A repeated-measures ANOVA revealed no significant difference in log response time during shape recognition as a function of color pairing, *F*(1.88, 249.76) = 0.70, *p* = 0.46.

#### Object free recall

Finally, we evaluated free recall for the objects as a function of the congruence conditions (Fig. 7C). A repeated-measures ANOVA revealed no significant difference in the number of objects freely recalled based on congruence condition, *F*(2, 270) = 0.64, *p* = 0.53.

### Discussion

Contrary to our predictions, we did not find better recognition memory for object shapes (i.e., an expectation-irrelevant feature) in the strong incongruent condition compared with the others. Instead, we found a significant difference in memory for object shape that was driven by the difference between the strong and weak congruent conditions. We also did not find a difference in response time and free recall across the different congruence conditions.

This suggests that enhanced memory for incidental details of incongruent events compared with other congruence conditions might not always extend to expectation-irrelevant object features. One caveat is that in the task, participants were only told to memorize the object and not necessarily the color or shape. As such, it is possible that during encoding participants prioritized shape as much as color. In this way, although the shape was irrelevant to the object–color expectation, it may have not been as incidental to memory as other features of events. Future work is needed to explore the impact of expectation-congruence on other kinds of expectation-irrelevant, incidental features. We return to this point in the discussion.

## General discussion

Recent studies have aimed to reconcile paradoxical findings of enhanced event memory for both expectation-congruent and expectation-incongruent events (Greve et al., 2019; Quent et al., 2022; Sherman & Frost, 2000; van Kesteren et al., 2012). One such account is the SLIMM framework (van Kesteren et al., 2012), which makes behavioral predictions for how expectation-congruence interacts with and influences event memory. In this paper, we explored whether the predictions of the SLIMM account extend to memory for the intrinsic features of objects. Across three experiments, we evaluated the potential non-linear relationship (e.g., U-shape) between congruence and memory, posited by SLIMM, in both recall and recognition. We also assessed how congruence impacts memory for incidental event details. Specifically, we manipulated the degree to which object features adhered to people’s prior expectations (e.g., object colors) and then assessed memory (recall and recognition) for both expectation-relevant (e.g., object color) and expectation-irrelevant features (e.g., object shape). We also assessed free recall of object names to further probe differences in memory resulting from color congruence. 

While past research has examined the impact of expectation-congruence on recall and recognition of associative pairs, a critical gap in the literature regarded whether the impact of expectation-congruence extends to object featural memory. Prior studies that have assessed the effect of congruence on object featural memory specifically compared memory for highly congruent and less congruent object features, but not highly incongruent object features (Hemmer & Steyvers, 2009; Persaud et al., 2017). As such, using a combination of studies and memory paradigms, here we sought to provide an in-depth examination of how varying levels of expectation congruence influences episodic memory for object features.

Study 1 examined the impact of object–color congruency on recall processes. We manipulated whether the color of a studied object matched people’s expectations and assessed memory for the object’s color using a cued-recall task. Contrary to our initial prediction, we did not observe a U-shaped pattern in recall as a function of expectation congruence. Instead, recall was only better for object features that were highly congruent with participants’ expectations compared with those that were highly incongruent or had no congruence. It appeared that congruency facilitated more accurate recall by helping participants stay within the correct studied category, reducing memory search time (as evidenced by faster response times), and lowering overall recall error.

One potential explanation for poorer memory performance in the incongruent condition is that memory search processes involved in recall might have been more effective at retrieving information that aligned with expectations rather than information that contradicted them (Sherman & Frost, 2000; Watkins & Gardiner, 1979). As such, the retrieval of congruent information could have masked how well incongruent information was persevered in memory. In fact, the retrieval impairment for object features in the incongruent condition was so severe that performance was statistically worse for incongruent object features than features with no congruence. In other words, for recall it was better to have no expectation than to have the wrong expectation when retrieving object features from memory. This seems to suggest that the recall impairment in retrieval for incongruent items was not solely attributed to or simply masked by the ease of retrieving congruent information. If this were the case, we would expect better memory for congruent compared with incongruent object features, but not better memory for the no congruence items compared with incongruent object features.

One way we sought to disentangle the impact of memory search and retrieval issues for incongruent items compared with the others was to look at errors for responses that fell within the correct color category. Here, we still observed an advantage for expectation-congruent items; however, the advantage for no congruence over incongruent items was no longer significant. To this end, future work is needed to investigate the basis for the retrieval impairment of incongruent items compared with other congruence conditions in recall. On one hand, it is likely that the retrieval impairment results from insufficient memory search processes for tagging incongruent items. On the other hand, the impairment might be exacerbated by the dual representation in memory that is created when individuals encounter expectation-incongruent events (e.g., I represent strawberries as red, but I just studied a blue strawberry). That is, when an individual encounters an expectation-incongruent item, they need to maintain two representations of that item in mind. The first is a representation based on their prior beliefs and experiences with that item (e.g., prior experiences with red strawberries) and another based on the current experience (e.g., studying a blue strawberry). Given the statistical regularities of the environment whereby expectation congruent items (e.g., red strawberries) are encountered, individuals should hold this information in mind for later real-world use. Yet, in a memory study, such as those detailed here, the individual also needs to hold the expectation-incongruent (e.g., blue strawberry) item in mind for subsequent memory tests. The need to maintain two representations of an object in mind, particularly in the incongruent condition, might be cognitively taxing. As such, it is possible that the dual representation creates interference during retrieval of incongruent items which, in turn, impairs recall compared with other conditions. Importantly, encountering expectation-congruent information does not create a dual representation problem in the same way, given that prior experiences cohere with the to-be-remembered congruent information.

In the present paradigm, we further examined congruency and memory utilizing a retrieval test that was less dependent on memory search processes: recognition memory. While previous research has also explored the impact of expectation-congruence on memory recognition, many studies have employed old/new recognition judgments as a memory measure (Cycowicz et al., 2008; Greve et al., 2019; Lew & Howe, 2017; Sherman & Frost, 2000; van Kesteren et al., 2013). Yet some past research has advocated for the use of forced-choice measures over old–new recognition tests given that old–new judgments are based not only on memory strength, but also subjective criterion to endorse items as old (Jang et al., 2009). Also, forced-choice designs are reportedly easier for participants to perform accurately (Macmillan & Creelman, 2005). As such, in the current study, we built on the existing literature by probing the impact of congruence on memory using a 4 AFC recognition design.

Study 2 investigated whether object–color congruency influenced recognition processes. Given that recognition processes are less contingent on memory search compared with recall, we predicted that participants would exhibit better memory for incongruent and congruent object–color items compared with no congruence items. However, we did not find any differences in recognition memory across the congruence conditions. Instead, all objects, regardless of their congruence condition, were remembered equally well in both recognition and free recall tasks. Although we did not observe differences in memory accuracy, we speculated that we might observe differences in the types of recognition response errors individuals made as a function of expectation-congruence. For example, if it is the case that participants have stronger memory traces for colors in the congruent and incongruent conditions compared with the no congruence condition, even when they are incorrect, we might expect them to choose options that are at least within the correct color category. However, if participants misremember object–color associations in the congruent and incongruent conditions, we might expect them to guess with their prior expectation. For the congruent condition, this would still lead to a higher proportion of within-category errors. However, in the incongruent condition, this would lead to a higher proportion of out-of-category errors. In contrast, we would expect random incorrect responses in the no congruence condition as there is no a priori color expectation to bias responses. Therefore, evaluating the errors in recognition responses might reveal differing underlying strategies that participants engage when recognizing congruent, incongruent, and no congruence associations.

To this end, we explored whether expectation congruence and response type shaped the kinds of errors participants made. We found that while congruence conditions did not influence the proportion of incorrect response types, there were significant differences in response types overall. That is, when participants were incorrect, they chose the option within the correct color category significantly more than the out of category options. Interestingly, we observed a marginal but nonsignificant interaction where participants were slightly more likely to choose an incorrect option within the correct color category in the congruent condition compared with the incongruent and no congruence conditions. This pattern of results has potentially different implications for the representation of each type of congruence item in memory. For the incongruent and no congruence conditions, individuals appeared to have encoded some episodic trace of the study event that allowed them to at least stay within the correct category. However, for the congruent condition, either individuals also encoded an episodic trace that allowed them to stay within the correct category, or they guessed with their prior expectation. Given the current design, it was impossible to disentangle these two possibilities, but future research may be able to elucidate the underlying representation that guides recognition memory across congruence conditions.

While the SLIMM account might explain the lack of difference between highly congruent and highly incongruent objects in Study 2, it did not explain the comparable performance in the no congruence condition. There were several possibilities that might explain this result. First, it is possible that the incongruence advantage might rely not only on the object–feature incongruence, but also the distinctiveness of incongruent items from others on the list. For instance, previous studies varied the distribution of incongruent and congruent objects (i.e., 50/50% vs. 75/25% congruent) and observed increased memory accuracy for incongruent items when the distribution of studied items consisted of more congruent object–color pairs (Morita & Kombara, 2022). The fewer items on the study list that were expectation-incongruent, the more distinct they were, thereby enhancing memory. In Study 2, participants studied an equal distribution of congruent, incongruent and no congruence object–color pairs which could have decreased the distinctiveness of the incongruent items, leading to comparable recognition memory.

A second and related possibility for not finding a difference across conditions is that although participants had clear color–object expectations, those expectations might not have been activated during encoding of the stimuli. Past work suggests that prior knowledge and expectations must be both activated and relevant in order to boost the learning and memory of new information (Brod, 2021). This point is illuminated in foundational work conducted by Bransford and Johnson (1972) which highlights the impact priming (activation of prior knowledge) has on an individuals’ ability to accurately recall and comprehend information. Thus, although participants may have had expectations about the objects in our study, these expectations might not have been properly activated and therefore may not have strongly influenced the encoding process. This undoubtedly would have consequences for retrieval, warranting future research.

While Studies 1 and 2 directly tested memory for expectation-relevant object features, Study 3 examined memory for object-shape, which was considered expectation-irrelevant (i.e., unrelated to the color congruence manipulation) in the current experimental context. According to the SLIMM account, the expectation-incongruent condition should produce enhanced memory for expectation irrelevant, incidental features of studied events compared with congruent conditions. The rationale for encoding incidental details is that they may help explain why the violation of expectation occurred and present an opportunity to update existing expectations. Contrary to this prediction, we did not find better shape recognition memory for objects in the strong incongruent condition compared with the other congruent conditions. Instead, we found better shape memory for objects paired with strong versus weakly congruent colors. There are two potential explanations for this result. First, the enhanced memory for strong-congruent colors, which seems to be assisted by prior knowledge, may have freed up individuals’ resources to encode additional information like the object shape. In contrast, the mismatch between prior expectations in the weak-congruent and strong-incongruent conditions may have taxed memory resources, making it difficult to encode shape with equal fidelity and effectively recognize shape from memory.

Relatedly, since object shape was irrelevant to the color-expectation manipulation, it was possible that this feature did not contain the adaptive value necessary to help explain the expectation incongruence. That is, prioritizing shape information did not provide sufficient information to help participants understand and process the weak congruent and strong incongruent colors (e.g., orangish-yellow bananas, purple pumpkins) to promote memory. Indeed, past work has found that expectation-incongruent information can be prioritized in memory when this information provides functional or adaptive value, such as helping individuals learn underlying categories (Sakamoto & Love, 2004) or informing where objects are found in natural scenes (Quent et al., 2022; but also see Ramey et al., 2022). In these contexts, the incongruent information possesses functional value allowing individuals to carry out a particular cognitive task, which in turn, might better situate this information in memory. Yet, in all three current studies, participants passively viewed weak congruent and strong incongruent items, which might have hampered the encoding and retrieval of the features of these items, expectation-relevant or otherwise. Future research is needed to explore how the functional relevance of expectation congruence impacts memory for objects and their constituent features. Specifically, this work might explore what kinds of incidental features are prioritized in memory for the purposes of reconciling prediction error and making sense of expectation-incongruent scenarios.

Overall, the results from this set of studies lend support for the *integration-elaboration* account of memory which suggests that expectation-congruent information is more easily elaborated on during encoding and, in turn, better retrieved at test. Consistent with Bein et al. (2015), we found that the enhanced memory for expectation-congruent information compared with other congruence conditions can also extend to featural information presented within expectation-congruent contexts. This extension might be facilitated by the fact that in expectation-congruent contexts, prior knowledge is more easily activated and deemed relevant to target items, assisting the encoding and retrieval of this information from memory (Bransford & Johnson, 1972). The current experimental design may not have facilitated the activation of prior knowledge for incongruent items, thereby hindering memory for incongruent objects and features. Taken together the current research, along with past work that has found mixed findings, highlights the fact that interactions between prior knowledge, expectation congruence, and memory are highly nuanced and complex, and further examination is needed to carve out a consistent and replicable account of how prior knowledge and expectations shape episodic memory.

## Limitations

While much of the findings in the present study were consistent with past research on interactions between congruence and memory, there were a number of limitations. First, the data from the three studies were collected immediately before, during, and immediately after the COVID-19 pandemic. As such, the data were collected in various modalities: Study 1 was conducted in person in a laboratory, while Studies 2 and 3 were conducted online—one moderated and the other unmoderated. Past research has raised similar concerns regarding differences in data collected in-lab versus online, finding that although both modalities can produce comparable results for certain memory tasks, online data tends to have greater variability in performance (Segen et al., 2021). Second, across all three studies, participants received different forms of compensation (e.g., course credit versus financial payment) which could have also resulted in differences in performance across the three studies. Third, while Studies 1 and 2 were conducted with psychology undergraduate students, Study 3 was run online with a slightly older participant sample (*M* = 38.5 years, *SD* = 11.04). Despite these differences, the goal of this work was to assess memory differences within studies, yielding interesting effects of color congruence within the different memory modalities tested. Nevertheless, although similar free recall results were observed across all three studies, direct comparisons should be made with caution.

Finally, the color-expectation conditions were based on a pilot study with a separate group of participants from those who took part in the memory studies. As such, it is possible that the memory participants’ slightly differed in their expectations for what the exact colors of the objects should be. This warrants caution in interpreting differences between the strong and weak congruence conditions. However, performance differences between those conditions were robust with significant recall error differences overall and within the correct category (Study 1) and shape recognition differences (Study 3). Nevertheless, future studies might consider a fully within subjects design where expectations are based on those of the memory participant. Finally, the congruence conditions slightly differed across the three studies, making a direct comparison across all four congruence conditions (i.e., strong congruent, weak congruent, strong incongruent, and no congruence) in color recall, color recognition, and shape recognition not possible. As such, future work should examine each memory process using more closely matched stimuli.

## Conclusion

Taken together, the findings across three studies shed light on how expectation-congruence impacts memory for the color and shape features of visual objects. While much of the evidence provides strong support for better memory for expectation-congruent object features, similar support for expectation-incongruent information was not observed. As such, there appears to be limits on the degree to which prevailing theories of event memory extend to memory for the features of objects. More research is needed to further examine interactions between congruence and object memory. Finally, understanding how congruence shapes the storage and retrieval of objects in memory has important implications for real world scenarios where object memory is brought to bear.

## Supplementary Information

Below is the link to the electronic supplementary material.

Supplementary file1 (DOCX 97 KB)

## Data Availability

The study stimuli and data from all three studies are publicly available on OSF upon publication: https://osf.io/5r7zj/?view_only=8975912e2e9c426e9c9b6b0da49acbd2

## Footnotes

1 Many of the objects that belonged to the same color category (e.g., strawberry, stop sign, Elmo) had nearly identical (not perceptually different) mean expected colors. Furthermore, the expected colors were similar to the mean color of the overall color category learned from previous studies (Persaud et al., 2021). This might have been a consequence of using the winHSL color space and holding saturation and luminance constant. Thus, to simplify the stimuli design, we derived one expected color for all objects within the same category by averaging over the means (with the exception of objects from the blue color category which some were expected to be darker blue, and others were expected to be light blue).

2 The recognition data reported here were part of a larger data set that also assessed long-term recognition memory. The data reported here are based on memory for the first day of the long-term memory study. See Macias and Persaud (2023) for long-term memory performance.

3 The object-color pairs in the congruent condition are akin to the weak congruence object color pairings in Study 1 and Study 3. Since we dropped the strong congruent condition in Study 2, we removed the adjectives “strong” and “weak” and simply labeled objects that align with a priori color expectations as items within the congruent condition.

4 For a visual of the response options by condition, refer to Fig. 3. The left panel of Fig. 3 describes the four response options in the congruent condition and the right panel of Fig. 3 describes response options in the incongruent condition. Importantly, the true expected (or prototypical color) for each object were distractors in both the congruent and incongruent conditions. For the congruent condition, it was the in-category distractor and for the incongruent condition, it was out-of-category distractor 2. For no congruence, the in-category distractor was always a color within the same color category as the target color and both out-of-category distractors were from a non-neighboring color category. The percentage of errors is given in the parentheses.

5 While both color and shape are useful for determining whether an object is expectation-congruent or incongruent, in this study, the shapes of the object were not manipulated to vary in congruence. Thus, shape was deemed an expectation-irrelevant object feature.

## Code availability

The analysis scripts are also publicly available on OSF.
