# Supplementary material for: Expectation-[in]congruence differentially impacts recall and recognition of object features
Source: Mem Cognit. 2025 Jun 10;54(1):85–108. doi: 10.3758/s13421-025-01740-x (PMC12864325; doi:10.3758/s13421-025-01740-x)
Supplement: Supplementary file 1 — Supplementary file1 (DOCX 97 KB) [file 13421_2025_1740_MOESM1_ESM.docx]

**Online Supplementary Materials**

Expectation-[In]congruence Differentially Impacts Recall and Recognition of Object Features

Kimele Persaud, Carla Macias, and Elizabeth Bonawitz

***Additional Analysis***

**1. Linear Mixed Effects Models**

In the main text, we implement anovas and planned contrasts to compare performance across color congruence conditions. However, it is likely that some of the variability in recall and recognition accuracy might be attributed to random effects of subject and items. As such, we implement mixed effects models on the main analyses across the three experiments to test whether the effects of congruence hold when accounting for random effects. The full models included memory accuracy, error, or response time as outcome measures, color congruence as a fixed effect, and subject and object as random effects. The models were run in R version 4.3.3 using the lme4 package (Bates et al, 2015).

**1a. Experiment 1: Color Recall Error**

Three linear mixed effects models were implemented to evaluate: 1) overall recall error, 2) within category recall error, and 3) response time. For each outcome variable we compared the full specified above to a reduced model that included the random effects only. For overall recall error, a likelihood-ratio test revealed that the full model which included an effect of color congruence provided a better fit to the data than a model that only included random effects of subject and item x^2^(3)=165.67, p<.0001. Relative to the strong congruent condition, the weak congruent condition was associated with a 74.9% rate of change in error (SE = .092, *t*=6.07), the strong incongruent condition was associated with a 233.5% rate of change in error (SE = .092, *t*=13.03), and no congruence condition was associated with a 146.1% rate of change in error (SE = .13, *t*=6.92).

For within category recall error, the full model also provided a better fit x^2^(3)=71.98, p<.0001. Relative to the strong congruent condition, the weak congruent condition was associated with a 4.24 increase in recall error (SE = .88, *t*=4.78), the strong incongruent condition was associated with a 8.04 increase in error (SE = .96, *t*=8.40), and no congruence condition was associated with a 5.71 increase in error (SE = 1.32, *t*=4.32).

Finally for response time, the full model provided continued to provide the superior fit x^2^(3)=58.72, p<.0001. Relative to the strong congruent condition, the weak congruent condition was associated with a 10.2% rate of change in seconds (SE = .027, *t*=3.62), the strong incongruent condition was associated with a 22.9% rate of change (SE = .027, *t*=7.62), and no congruence condition was associated with a 14.6% rate of change (SE = .03, *t*=4.58).

**1b. Experiment 2: Color Recognition Accuracy**

Two linear mixed-effects models (one generalized for binary dependent variable) were implemented to evaluate 1) recognition accuracy and 2) response time. We compared the full model specified above for each outcome variable to a reduced model with just random effects. Consistent with the main analysis in the paper, there was no difference in the model fits for recognition accuracy, x^2^(1)= 0.30, p= 0.58. Similarly, we found no difference in model fit for response time, x^2^(1)= 0.003, p= 0.96.

**1c. Experiment 3: Shape Recognition Accuracy**

Two linear mixed effects models (one generalized for binary dependent variable) were implemented to evaluate the effect of color congruence on shape recognition accuracy and response time. Similar to studies 1 and 2, we compared the full specified to a reduced model that included the random effects only for each outcome variable. For recognition shape accuracy, a likelihood-ratio test revealed that the full model which included an effect of color congruence provided a marginally, non-significant better fit to the data than a model that only included random effects of subject and item x^2^(2)=4.92, p=.085. Relative to the strong congruent condition, the weak congruent condition was associated with a 31.50% lower rate of change in recognition accuracy (SE = .17, *z=*-2.22, *p*=.03), but no difference from the strong incongruent condition (SE = .17, *z=*-0.83, *p*=.41).

Since the difference between the full and reduced models was only marginal, we do not draw strong conclusions regarding the difference between the strong and weak congruent condition. Note that study 3 was run unmoderated online and past research suggests that online data produces greater variability in performance and may require increased sample sizes to achieve comparable performance to studies in the lab (Segen et. al.,2021). Although we did increase the number of participants, we substantially reduced the number of trials. As such, future work is needed to further explore the impact of expectation-congruence on recognition of expectation incidental features.

For response time, there was no difference in fit between the full model and the reduced model (x^2^(2)=.81, p=.67), suggesting that the congruence had no effect on how long it took participants to recognize object shapes.

**2. Pilot Results**

The pilot study described in the main document was implemented to evaluate the mean expected color and the standard deviation around the expected color for each object. Below we provide a table that contains this information for each object. Note that as expected, the objects unassociated with strong color expectations (e.g., t-shirt, book, etc.) have substantially larger standard deviations than the objects associated with expected color.

| **Object** | **Mean (range 0-239)** | **SD** |
| --- | --- | --- |
| **Strawberry** | 236.31 | 5.34 |
| **Stop sign** | 238.73 | 4.2 |
| **Pumpkin** | 20.01 | 2.98 |
| **Banana** | 38.44 | 1.43 |
| **Broccoli** | 85.57 | 9.76 |
| **Tear drop** | 125.82 | 4.42 |
| **Jeans** | 144.92 | 11.35 |
| **Eggplant** | 178.25 | 3.77 |
| **Pig** | 210.58 | 9.06 |
| **Elmo** | 238.23 | 3.22 |
| **Tigger** | 19.77 | 3.75 |
| **SpongeBob** | 38.24 | 1.43 |
| **Pikachu** | 37.48 | 3.23 |
| **Grinch** | 77.27 | 10.22 |
| **Kermit** | 81.28 | 8.43 |
| **Cookie Monster** | 147.17 | 9.64 |
| **Genie** | 140.58 | 15.51 |
| **Patrick Star** | 209.01 | 7.41 |
| **Fish** | 98.40 | 58.36 |
| **Butterfly** | 117.70 | 72.31 |
| **Umbrella** | 129.05 | 74.54 |
| **Toothbrush** | 126.79 | 52.73 |
| **Book** | 85.46 | 74.20 |
| **Shirt** | 149.53 | 65.55 |
| **Flower** | 98.11 | 82.18 |
| **Lego** | 76.27 | 76.25 |
| **Car** | 148.17 | 71.01 |

**3. Position Analysis**

**3a. Experiment 1: Color Recall**

Based on the results of better recall for congruent compared to incongruent items, we speculated that the mechanism for searching memory is better able to tag congruent versus incongruent items. As such, we investigated whether this search benefit influenced the order in which the objects based on color congruence were recalled. We might expect that expectation-congruent items are recalled earlier on in the retrieval process compared to incongruent and no congruent objects. To assess this possibility, we explored whether there were differences in the number of strong and weak congruent, strong incongruent, and no congruence items recalled within the first ten instances of remembering object labels. Overall, no strong discernible pattern emerged between the strong congruent and incongruent items. Based on visual inspection, there doesn’t appear to be clear differences in the order of recall for the strong congruent and strong incongruent conditions. Interestingly, however, it does appear that participants recalled no congruent items fairly consistently across output positions. Also, the weak congruent items appear to be recalled earlier on and then drop off in recall at later output positions.

**Figure 1**

*Recall Position of Objects based on Color Congruence in the Color Recall Study*


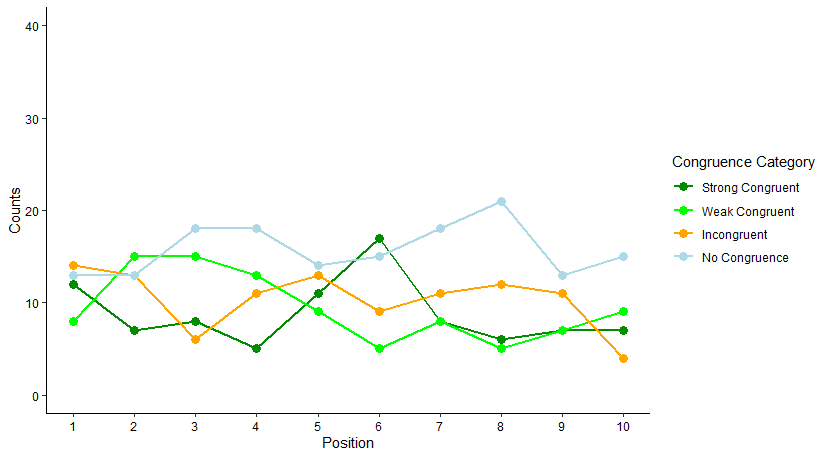


**3b. Experiment 2: Recognition of Object-Color Pairs**

In Experiment 2, we also explored whether there were differences in the number of Congruent, Incongruent, and No Congruence objects recalled within the first ten instances of remembering object labels. If there was a clear pattern of congruent, incongruent, or no congruence objects recalled more frequently early on, this may suggest that those objects were more readily accessible during recall. Overall, we found that participants consistently recalled congruent objects more frequently and earlier on relative to incongruent objects. Interestingly, we also found that no congruent objects were recalled frequently early on. One potential explanation for this is that no congruent condition contained objects that were common, highly familiar, and frequently used in the English language (e.g., shirt, butterfly, book, etc.). Although participants were familiar with the expectation-related objects, some objects might have been less common. Taken together, the results from Experiment 1 and 2 suggest that congruence could potentially play a role in serial recall, but more work is needed to examine the relationship between congruence and the output positions in which objects are recalled.

**Figure 2**

*Recall Position of Objects based on Color Congruence in the Color Recognition Study*

*
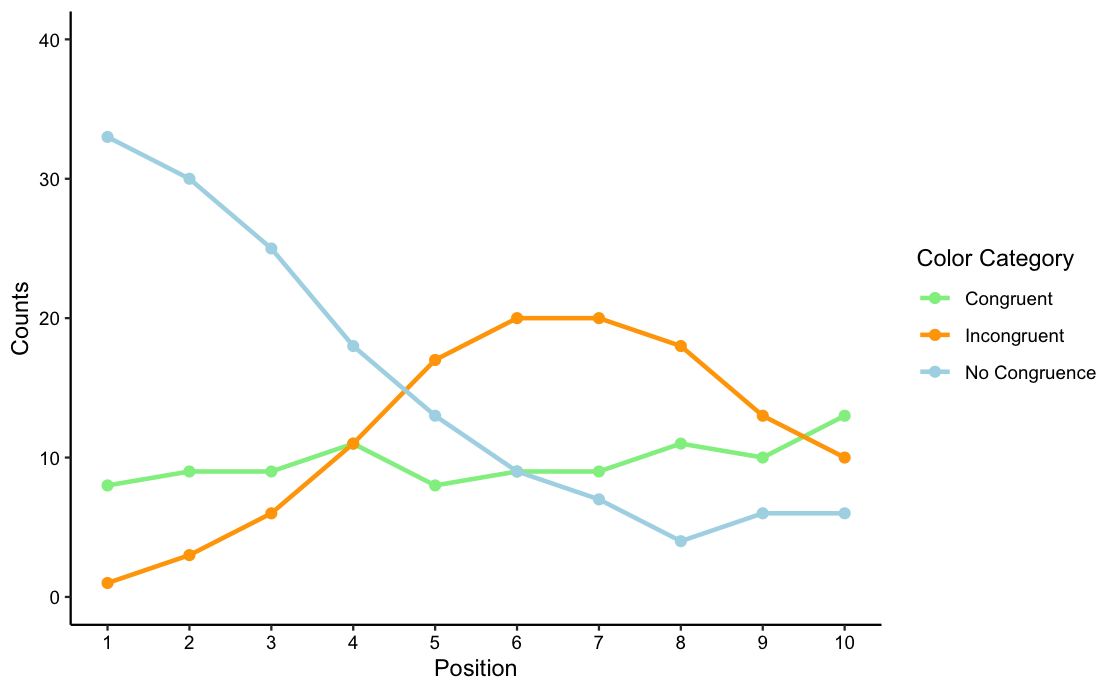
*
